# Périartérite noueuse: une étiologie de myélite à ne pas méconnaître en Afrique subsaharienne

**DOI:** 10.48327/mtsi.v5i4.2025.789

**Published:** 2025-12-19

**Authors:** Josaphat IBA BA, Annick MFOUMOU, Arthur KANGANGA EKOMY, Aurélie BADERHWA, Armel TCHIBENET RAPONTCHOMBO, Michel Arnaud SAPHOU DAMON, Léonie Esther LEDAGA LENTOMBO, Marielle IGALA, Jean Bruno BOGUIKOUMA

**Affiliations:** 1 Service de médecine interne, Centre hospitalier universitaire de Libreville, BP 2228, Libreville, Gabon; 2 Service de réanimation, Clinique Chambrier, BP 2230, Libreville, Gabon; 3 Service de neurologie, Centre hospitalier universitaire de Libreville, BP 2228, Libreville, Gabon Auteur correspondant: ibabajose@yahoo.fr

**Keywords:** Périartérite noueuse, Anticoagulants du lupus, Myélite transverse, ANCA, Gabon, Afrique subsaharienne, Polyarteritis nodosa, Lupus anticoagulants, Transverse myelitis, ANCAs, Gabon, Sub-Saharan Africa

## Abstract

**Introduction:**

La périartérite noueuse (PAN) est une vascularite segmentaire nécrosante systémique intéressant les artères de moyen ou de petit calibre. Elle se caractérise par une multiplicité de signes cliniques secondaires à une infiltration des différentes couches de la paroi artérielle (adventice, média, intima) par des neutrophiles, des lymphocytes, et des éosinophiles. Cette infiltration cellulaire est responsable d’une réduction du calibre artériel de minime à sévère, à l’origine de manifestations surtout neurologiques et digestives. Ces manifestations se compliquent d’une altération de l’état général, source d’errances diagnostiques en Afrique subsaharienne avec certaines maladies infectieuses endémiques (VIH/sida, tuberculose et hépatites virales). Nous rapportons deux observations de PAN avec atteinte neurologique périphérique inaugurale (paraplégie et tétraplégie), secondaire à une myélite transverse documentée, chez deux patientes ayant des antécédents obstétricaux de pertes fœtales. À travers ces deux observations, nous voulons préciser les caractéristiques de cette association (PAN et anticorps antiphospholipides) peu décrite.

**Observations:**

Les patientes étaient âgées de 25 et de 45 ans, et avaient en commun la conjonction de migraine chronique, fausses couches, déficit neurologique périphérique majeur, survenant en saison sèche (saison de basses températures au Gabon), myélite transverse documentée à l’imagerie par résonnance magnétique, et dont le bilan auto-immun exhaustif ne retrouvait qu’une positivité de l’anticoagulant circulant du lupus.

**Discussion:**

Si le diagnostic de périartérite noueuse était établi chez ces deux patientes, on peut discuter du rôle isolé des anticoagulants du lupus. En effet, ces anticorps antiphospholipides sont susceptibles, par leur persistance dans le sérum et cela même en l’absence d’activité de la maladie, de créer les conditions de vascularite.

**Conclusion:**

L’association corticothérapie/immunosuppresseur/kinésithérapie intensive permettait une récupération neurologique satisfaisante à six et sept mois.

## Abstract (en)

**Introduction:**

Polyarteritis nodosa (PAN) is a systemic necrotizing segmental vasculitis that affects medium-and small-caliber arteries. It is characterized by infiltration of the different layers of the arterial wall (adventitia, media, and intima) by neutrophils, lymphocytes, and eosinophils, resulting in a variety of clinical signs. This infiltration is responsible for reducing arterial caliber, which can range from minimal to severe and cause neurological and digestive manifestations. These manifestations are complicated by a deterioration in the patient’s general condition, leading to diagnostic uncertainty in sub-Saharan Africa, where certain infectious diseases (e.g., HIV/AIDS, tuberculosis, and viral hepatitis) are endemic. We present two cases of PAN with initial peripheral neurological involvement (paraplegia and quadriplegia) secondary to transverse myelitis in patients with a history of fetal loss. Through these two case studies, we aim to clarify the characteristics of this rarely described association between PAN and antiphospholipid antibodies.

**Case reports:**

The patients, aged 25 and 45, presented with chronic migraines, miscarriages, and a major peripheral neurological deficit that occurred during the dry season (the low-temperature season in Gabon). Magnetic resonance imaging revealed transverse myelitis. An exhaustive autoimmune assessment yielded only a positive result for circulating lupus anticoagulant.

**Discussion:**

Although we diagnosed polyarteritis nodosa in these two patients, the role of the lupus anticoagulants remains debatable. These antiphospholipid antibodies are likely to create the conditions for vasculitis due to its persistence in the serum even in the absence of disease activity.

**Conclusion:**

The combination of corticosteroid therapy, immunosuppressants, and intensive physical therapy resulted in satisfactory neurological recovery at six and seven months.

## Introduction

La périartérite noueuse (PAN) est une vascularite segmentaire focale nécrosante affectant les artères de calibre moyen (artères rénales, hépatique, coronaires, cœliaque et mésentérique) ou petit (artères intraparenchymateuses) sans glomérulonéphrite ni vascularite des artérioles, des veinules ou des capillaires [3]. La première observation a été rapportée par Kussmaul et Maïer en 1866, correspondant sur le plan histologique à une panartérite avec infiltration de l’adventice par des polynucléaires neutrophiles et des lymphocytes, une nécrose fibrinoïde de la média, et une prolifération endothéliale de l’intima. Sur le moyen et/ou long terme peuvent apparaitre des anomalies de la paroi vasculaire à type de microanévrisme, de sténose, et/ou de thrombose *in situ.* Les manifestations cliniques diverses qui en découlent dépendent des territoires et organes irrigués ou drainés par ces vaisseaux. Le caractère systémique de cette affection suscite de multiples diagnostics différentiels, et fait qu’elle reste encore mal connue des praticiens d’Afrique subsaharienne. L’atteinte neurologique représenterait 50 à 75% des atteintes au cours de la PAN avec de façon prédominante une atteinte du système nerveux périphérique [2]. Nous rapportons deux observations avec déficit majeur: l’un à type de tétraplégie et l’autre de paraplégie, réversible sous traitement spécifique.

## Observation 1

**Figure 1:**
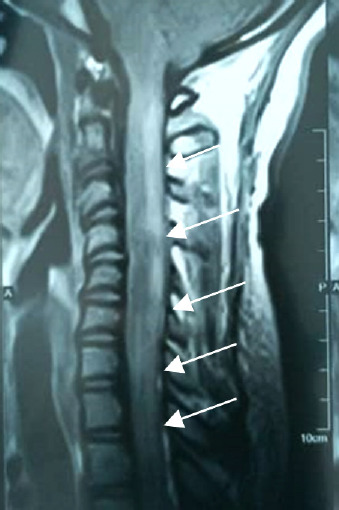
Élargissement et agrandissement de la moelle spinale au niveau cervico-thoracique jusqu’à T5, de topographie centro-médullaire mesurant 12x8 de diamètres axiaux, étendue sur 201mm de hauteur (IRM Séquence T2 coupe sagittale)

Mademoiselle BMDF, gabonaise sans emploi, âgée de 25 ans consulte en janvier 2025. Elle signale des antécédents essentiellement obstétricaux (une fausse couche dans le premier trimestre, un accouchement prématuré à 24 semaines d’aménorrhée avec naissance d’une fille âgée actuellement de 2 ans). Elle présente 3 ou 4 jours après un épisode fébrile, une douleur abdominale invalidante avec arrêt des matières et des gaz, suivie de rétention d’urine et de paraplégie brutale, motivant son hospitalisation dans le service de médecine interne. Avant cet épisode, elle avait successivement présenté pendant huit à neuf mois des épisodes de migraine à bascule difficilement contrôlable par du paracétamol et des douleurs des extrémités des mains et des pieds. Cependant, ces troubles ne s’accompagnaient pas de myalgie, de syndrome de Raynaud, de syndrome sec oculo-buccal, ou de troubles de la vision. L’état général était conservé malgré une perte de poids de 5 kilogrammes sur trois mois. Elle était apyrétique, normotendue, et eupnéique. Les éléments pertinents cliniques se résumaient à une main tombante droite, une paraplégie avec troubles sphinctériens, et nouures des 2 faces internes des avant-bras. Sur le plan biologique, la protéine C réactive (CRP) était normale (< 6 mg/l). La vitesse de sédimentation n’a pas été réalisée. Les leucocytes étaient à 10 510/mm^3^ à prédominance neutrophiles, le taux d’hémoglobine à 11,7 g/dl, et les plaquettes normales. Le bilan rénal, hépatique, la glycémie, le cholestérol et ses sous-fractions, l’hormonologie thyroïdienne, la bandelette urinaire et le temps de céphaline activé (TCA) étaient normaux. Les sérologies hépatitiques B et C, VIH 1,2, et syphilitiques étaient négatives. Sur le plan immunologique, le test de Coombs et l’anticoagulant circulant du lupus étaient positifs, les anticorps anticardiolipine (IgM et IgG), antinucléaires, anti DNA natifs et anticytoplasmiques des neutrophiles (ANCA) négatifs. Sur le plan morphologique, la tomodensitométrie abdominopelvienne était normale, et l’IRM médullaire objectivait une myélite extensive jusqu’à la 5^e^ vertèbre thoracique mesurant 201 millimètres de hauteur (Fig. 1). La biopsie cutanée d’une lésion purpurique de la face postérieure de la cuisse, apparue à 48 heures de l’hospitalisation, retrouvait une vascularite leucocytoclasique. Sur la présence de 6 des 15 critères de Guillevin (Tableau I) (fièvre, altération de l’état général, paraplégie, nodules sous cutanés, arthralgies distales et douleur abdominale inaugurale), était retenu le diagnostic de périartérite noueuse. Sur le plan thérapeutique, la patiente était traitée par bolus de méthyl prednisolone à raison de 1 g/jour pendant 3 jours. Ce traitement a été relayé par de la prednisone à 1 mg/kg/jour (soit 50 mg/jour) et à des perfusions de cyclophosphamide de 1 gramme/mois pour un total de 6 cures, relayées par de l’azathioprine à 150 mg/jour, couplée à une kinésithérapie intensive. Ce trépied thérapeutique permettait à 5 mois la reprise de verticalisation de la patiente avec une autonomie de marche limitée aux gestes du quotidien, et à 7 mois d’une récupération optimale avec discrets troubles persistants de la sensibilité superficielle.

**Tableau I:** Critères de Guillevin

Altération de l’état général
Fièvre
Multinévrite
Syndrome inflammatoire
Cardiomyopathie primitive
Purpura infiltré et/ou nodules sous-cutanés
Polyarthrite
Myalgies
Artériopathie distale
Orchite non infectieuse
Néphropathie vasculaire et/ou glomérulaire
Crises douloureuses abdominales
Hypertension artérielle
Accident neurologique central
Asthme récent grave

Diagnostic = 5/15 critères (sensibilité de 70,6%, spécificité de 92,3%)

## Observation 2

**Figure 2:**
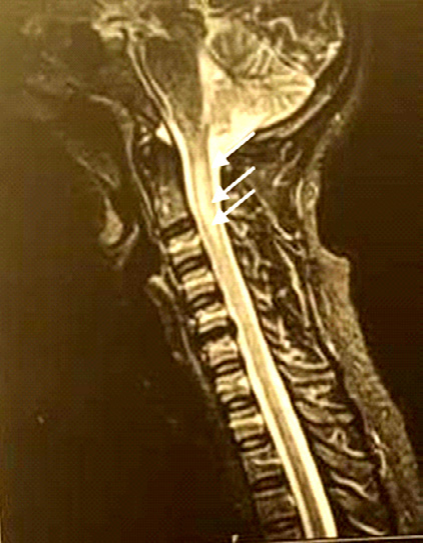
Hypersignal étendu centromédullaire de C2 à C6 (Séquence T2 coupe sagittale)

Mademoiselle OMC, de nationalité gabonaise, fonctionnaire, âgée de 45 ans, présentait en décembre 2023, des épisodes de myalgies des membres d’aggravation progressive, se compliquant à trois mois de tétraplégie. Avant l’installation de ce déficit de traumatisme, il n’existait pas de contexte fébrile ou grippal, de prise médicamenteuse ou de décoctions traditionnelles, ni de consommation d’alcool. Depuis 4 ans, elle présentait des épisodes d’asthénie invalidante spontanément régressive, avec douleur abdominale spasmodique postprandiale améliorée alternativement par antiinflammatoires non stéroïdiens et inhibiteur de la pompe à proton. Ses antécédents se résumaient à des épisodes de bleus spontanés des membres inférieurs, une migraine à bascule évoluant depuis près de 20 ans, et un épisode d’amaurose en novembre 2020 spontanément résolutif. Elle n’avait jamais été jusque-là hospitalisée ni opérée. Sur le plan obstétrical, elle rapportait 3 fausses couches spontanées dans le premier trimestre, n’avait pas d’enfants, et présentait depuis 2 mois une aménorrhée non gravidique. L’état général était conservé avec un indice de masse corporel de 23 kg/m^2^ malgré une perte de poids de 6 kg. Elle était apyrétique, normotendue et eupnéique. Les éléments de l’examen clinique se résumaient à une tétraplégie, avec des réflexes ostéotendineux, cutanéo-abdominaux et cutanéo-plantaires absents. Il existait des hématomes spontanés de la face antérieure du genou gauche et du tiers supérieur de la jambe, ainsi que de multiples nouures des membres inférieurs prédominant à la face interne des jambes et des cuisses. Sur le plan biologique les leucocytes étaient à 12 750/mm^3^ avec une prédominance neutrophile, le taux d’hémoglobine à 13,5 g/dl, et les plaquettes normales, la vitesse de sédimentation à 35 mm, la CRP à 98 mg/l. Créatine phosphokinase, ionogramme sanguin, glycémie à jeun, cholestérol et sous-fractions, calcémie, phosphorémie, transaminases, taux de prothrombine et temps de céphaline activée étaient normaux. La protéinurie était positive à une croix avec nitrites, sans hématurie, ni leucocytes. Les sérologies des virus hépatotropes (Ag Hbs et anticorps anti VHC) et HTLV1 étaient négatives; la sérologie *Borrelia burgdorferi* positive en IgG. La ponction lombaire ramenait un liquide clair normotendu paucicellulaire avec de rares macrophages, une absence de polynucléaires, de lymphocytes, et de cellules atypiques. Le test de Coombs (direct et indirect), les autoanticorps antinucléaires, anti DNA natifs, autoanticorps nucléaires solubles, anticardiolipine (IgM et IgG), anti-béta 2 glycoprotéine 1, ANCA, étaient négatifs. L’anticoagulant circulant du lupus était positif. L’IRM médullaire retrouvait une myélite transverse longitudinalement étendue de C2 à C6 (Fig. 2). La tomodensitométrie abdominopelvienne, et l’angioscanner rénal et des artères digestives étaient normaux. L’électromyogramme n’avait pu être réalisé faute de disponibilité. Avec la conjonction de 7 critères de Guillevin sur 15 (altération de l’état général, multinévrite, nodules sous cutanés, myalgies, crises douloureuses abdominales, néphropathie glomérulaire et syndrome inflammatoire), le diagnostic de périartérite noueuse était envisagé. Elle était traitée par bolus de méthylprednisolone (Solumédrol^®^) (960 mg/jour pendant 3 jours) relayés par une corticothérapie de 1 mg/kg/jour de prednisone (Cortancyl^®^) soit 80 mg/jour, perfusions de cyclophosphamide (Endoxan^®^) à raison de 1 g/mois pendant 6 mois suivi de relai oral, antiagrégant plaquettaire à la dose de 100 mg/ jour, rosuvastatine 20 mg/jour et kinésithérapie intensive. Devant la récupération lente, la patiente était évacuée, après trois mois de ce traitement, vers l’Europe, pour réalisation de plasmaphérèses et poursuite de la rééducation fonctionnelle. À six mois, elle retrouvait une autonomie des quatre membres.

## Discussion

La PAN demeure le prototype de vascularite nécrosante systémique. Il y a encore deux décennies, la PAN était rapportée en association avec l’hépatite B dans 36 à 50% des cas [2], affection sévissant à l’état endémique en Afrique subsaharienne. En 2020, l’Afrique recensait 26% des cas mondiaux d’hépatite B [11]. Paradoxalement, en tenant compte de ce contexte, on peut se poser la question de savoir si la PAN n’est pas sousdiagnostiquée en Afrique subsaharienne. Très souvent, il s’agit de cas uniques rapportés. Ndongo *et al.,* dans une étude au Sénégal portant sur 27 vascularites, retrouvait 2 PAN associées au virus de l’hépatite B (VHB) [9]. Le VHB au cours de la PAN induit une réaction de « type III » où les complexes immuns sont piégés dans les parois vasculaires, provoquant des lésions vasculaires [16,17]. Des complexes immuns antigènes Hbs et anticorps anti-Hbs ont été mis en évidence dans ces lésions vasculaires. Grâce à la vaccination contre le VHB, et l’amélioration des conditions d’hygiène, la fréquence de la PAN due au VHB est en voie de diminution passant de 30% à 7% [18]. En effet, nous retrouvons dans nos observations une négativité de l’antigène Hbs, sans remise en cause du diagnostic de PAN.

Nous avons retenu le diagnostic de PAN devant l’existence de 6 et 7 critères de Guillevin. Pour le diagnostic, nous n’avons pas choisi les critères ACR du fait de la difficulté à réaliser chez nos patientes un bilan artériographique à la recherche d’anévrismes et une biopsie d’une artère de petit ou moyen calibre à la recherche de polynucléaires dans la paroi artérielle. En outre, les critères de l’ACR présentent une moins bonne spécificité (86% contre 92,3%) (Tableau II).

**Tableau II:** Critères de l’American College of Rheumatology (ACR) 1990

Items	Valeur
Amaigrissement > 4 kg	1
Livedo reticularis	1
Douleur ou sensibilité testiculaire	1
Myalgies diffuses, faiblesse musculaire ou sensibilité des membres inférieurs	1
Mono-ou polyneuropathie	1
Pression diastolique > 90 mmHg	1
Insuffisance rénale (urée > 040 g/l ou créatininémie > 15 mg/l)	1
Marqueurs sériques de l’hépatite B (antigène HBs ou anticorps anti-HBs)	1
Anomalies artériographiques (anévrismes et/ou occlusions des artères viscérales)	1
Biopsie d’une artère de petit ou moyen calibre montrant la présence de polynucléaires dans la paroi artérielle	1

Diagnostic = 3/10 critères (sensibilité de 82,2%, spécificité de 86,6%

Nos patientes ont en commun une histoire de migraine, de fausses couches, et une positivité des anticoagulants circulant du lupus, qui constituent des stigmates du syndrome des anti-phospholipides (SAPL). Elles présentent également une myélite transverse avec déficit neurologique majeur (tétraplégie et paraplégie) et un contexte de survenue en saison sèche, saison où les températures sont les plus basses sur l’année au Gabon. Le SAPL se définit par l’association des signes obstétricaux et thrombotiques, couplés à la positivité d’anticorps antiphospholipides (anticoagulants circulant lupiques, anticorps anticardiolipine (IgM et IgG) et d’anticorps anti-β2 glycoprotéine-1 (IgM et IgG) [16]. Certains auteurs ont émis l’hypothèse que les anticorps antiphospholipides favoriseraient le développement d’une vascularite, déclenchée par une inflammation vasculaire révélant des antigènes cryptiques stimulant des anticorps anti-cellules endothéliales [5,10]. Chez certains patients, ces anticorps persisteraient dans le sérum, et ce même en l’absence d’activité de la maladie [15].

Les *vasa nervorum* qui assurent l’apport artériel du système nerveux périphérique, sont l’un des sites les plus fréquemment touchés au cours de la PAN. Ces *vasa nervorum* pénètrent dans le nerf et se divisent en réseaux microvasculaires anastomosés complexes qui passent en grande partie dans l’épinèvre, le tissu conjonctif (qui recouvre les nerfs périphériques) et comble l’espace interfasciculaire [1]. Cela pourrait expliquer pourquoi jusqu’à 85% des patients atteints de PAN développent une neuropathie périphérique de survenue brutale, avec un déficit complet, ou moins souvent partiel, à type de mononévrite, de multinévrite et de polyradiculonévrite ascendante [12]. Les anomalies du système nerveux central sont moins fréquentes que celles du système nerveux périphérique et ont tendance à survenir plus tardivement au cours de la maladie [7]. L’apparition rapide d’une paraparésie/paraplégie ou d’une tétraparésie/tétraplégie associée à un déficit sensitif bilatéral ayant un niveau supérieur précis et à des troubles sphinctériens, définit cliniquement les myélopathies aiguës transverses totales. Dans les myélopathies aiguës transverses partielles, l’atteinte médullaire est asymétrique, parfois unilatérale; le déficit sensitif peut être au premier plan. La réalisation d’une IRM médullaire en urgence est indispensable pour éliminer une myélopathie aiguë d’origine compressive [6]. La survenue de PAN en période de basse température sur l’année pourrait faire discuter dans notre étude de son rôle comme déclencheur dans l’expression de cette maladie. Cependant, aucun profil saisonnier n’a été observé pour cette vascularite dans la littérature [13].

Les étiologies des myélites transverses sont nombreuses, réparties en cause primitive et secondaires. Les premières sont responsable de 2/3 des atteintes. Les données génétiques déterminent la susceptibilité génétique, et les facteurs environnementaux déclenchent la maladie. Les causes secondaires sont responsables d’un tiers des cas. Elles comprennent les causes infectieuses, en particulier les causes virales telles que l’hépatite B et C, l’infection par le VIH, le parvovirus B19 et le virus d’Epstein-Barr dans sa forme active. Parmi les autres causes, on trouve des causes hématologiques telles que la leucémie à tricholeucocytes, la leucémie myélomonocytaire et le syndrome myélodysplasique. Les causes auto-immunes comprennent le lupus, le syndrome des antiphospholipides, la sclérose en plaques et la neuromyélite optique. Dans ce cas, un test de Coombs positif et un nombre suffisant de critères de Guillevin, nous a orienté vers une cause immunologique, conduisant au diagnostic de PAN. Nous avons constaté chez nos deux patientes une récupération neurologique lente ayant nécessité plusieurs mois en raison du caractère axonal des lésions. La récupération est souvent de bonne qualité mais parfois incomplète, avec sur le plan sensitif, des douleurs résiduelles pouvant persister comme chez une de nos patientes [14].

Des thérapies novatrices (biothérapies) ont été utilisées dans les cas de PAN récidivante ou réfractaire: tocilizumab, anti-TNF alpha et rituximab, permettant une rémission complète dans respectivement 50%, 40% et 33% des cas, avec des profils de sécurité comparables [4]. Au Gabon, l’assurance maladie nationale (encore appelée Caisse nationale d’assurance maladie et de garantie sociale) prend en charge 80% du coût effectif du médicament pour les affections courantes et 90% concernant les affections à longue durée [8]. La périartérite noueuse est la 21^e^/30, au même titre que le lupus systémique et la sclérodermie systémique [8]. Pour le rituximab qui est à ce jour la seule biothérapie prise en charge, le patient paie 145,3 €.

## Conclusion

La PAN est une vascularite nécrosante autoimmune très peu connue des praticiens africains, dont le pronostic peut être redoutable en l’absence de diagnostic. Il importe de vulgariser ce groupe d’affections dans les programmes universitaires des facultés de médecine d’Afrique subsaharienne, et de façon plus globale les maladies auto-immunes, étiquetées à tort par les praticiens d’Afrique subsaharienne, de « maladies des occidentaux ».

## Consentement des patientes

Nous avons obtenu le consentement des patientes pour la publication de cet article.

## Financement

Cette étude n’a reçu aucun financement.

## Contribution des auteurs et autrices

IBA BA Josaphat: concepteur et rédacteur. MFOUMOU Annick: relecteur et collecteur de données

KANGANGA EKOMY Arthur et Baderhwa Aurélie: recherche bibliographique, relecteurs

TCHIBENET RAPOTCHOMBO Armel et

SAPHOU DAMON Michel Arnaud: choix des iconographies, relecteurs

LEDAGA LENTOMBO Léonie Esther et IGALA Marielle: relecteurs

BOGUIKOUMA Jean Bruno: concepteur, relecteur, approbateur de la version finale

## Déclaration de liens d’intérêt

Aucun lien d’intérêt n’a été déclaré.
