# Supplementary material for: Polyarteritis nodosa: an etiology of myelitis that should not be ignored in subsaharansub-Saharan Africa
Source: Med Trop Sante Int. 2025 Dec 19;5(4):mtsi.v5i4.2025.789. [Article in French] doi: 10.48327/mtsi.v5i4.2025.789 (PMC12948370; doi:10.48327/mtsi.v5i4.2025.789)
Supplement: Related texts

# Research article: Polyarteritis nodosa: an etiology of myelitis that should not be ignored in subsaharansub-Saharan Africa

## Introduction

Polyarteritis nodosa (PAN) is a necrotizing vasculitis that affects medium-sized arteries, such as the renal, hepatic, coronary, celiac, and mesenteric arteries, as well as small arteries, such as intraparenchymal arteries. It is not associated with glomerulonephritis or vasculitis of the arterioles, venules, or capillaries [3]. Kussmaul and Maier first observed the condition in 1866. Histologically, it corresponds to panarteritis with neutrophil and lymphocyte infiltration of the adventitia, fibrinoid necrosis of the media, and endothelial proliferation of the intima. In the medium and/or long term, complications such as microaneurysms, stenosis, and/or thrombosis may occur in the vascular wall. The resulting clinical manifestations depend on the areas and organs supplied or drained by these vessels. The systemic nature of this condition leads to multiple differential diagnoses and means that it is poorly understood by practitioners in sub-Saharan Africa. Neurological involvement accounts for 50 to 75% of PAN cases, predominantly affecting the peripheral nervous system [2]. We present two cases involving significant deficits: one patient experienced quadriplegia, and the other experienced paraplegia. Both cases were reversible with specific treatment.

## Case 1

Ms. BMDF, a 25-year-old unemployed woman from Gabon with a medical history primarily involving obstetrics (one miscarriage in the first trimester and one premature delivery at 24 weeks of amenorrhea, resulting in the birth of a daughter who is now two years old), presented in January 2025. Three or four days after a feverish episode, she experienced debilitating abdominal pain, accompanied by an absence of bowel movements and gas. This was followed by urinary retention and sudden paraplegia, which led to her hospitalization in the internal medicine department. Prior to this episode, she had experienced migraines for eight to nine months that were difficult to control with paracetamol, as well as pain in her hands and feet. However, these symptoms were not accompanied by myalgia, Raynaud’s syndrome, dry eye or mouth syndrome, or vision problems. Despite a weight loss of five kilograms over three months, her general condition remained stable. She was afebrile, normotensive, and eupneic. The only relevant clinical findings were a drooping right hand, paraplegia with sphincter disorders, and nodules on the inner sides of both forearms. Biologically, C-reactive protein (CRP) was normal (below 6 mg/L), the sedimentation rate was not measured, and the following values were normal: leukocytes (10,510/mm^3^), hemoglobin (11.7 g/dL), and platelets. Renal and hepatic function tests, blood glucose, cholesterol and its subfractions, thyroid hormones, a urine strip test, and an activated partial thromboplastin time (APTT) test were normal. Hepatitis B and C, HIV 1 and 2, and syphilis serology tests were negative. Immunologically, the Coombs test and the circulating lupus anticoagulant test were positive. However, anticardiolipin antibodies (IgM and IgG), antinuclear antibodies, anti-native DNA antibodies, and antineutrophil cytoplasmic antibodies (ANCAs) were negative. Abdominal and pelvic CT scans were morphologically normal. Spinal MRI showed extensive myelitis extending to the fifth thoracic vertebra, measuring 201 millimeters in height (Fig. 1). A skin biopsy of a purpuric lesion on the posterior thigh, which appeared 48 hours after admission, revealed leu-kocytoclastic vasculitis. Based on the presence of six of the fifteen Guillevin criteria (Table I) (fever, altered general condition, paraplegia, subcutaneous nodules, distal arthralgia, and initial abdominal pain), a diagnosis of polyarteritis nodosa was made. The patient received a bolus of methylprednisolone at a dose of one gram per day for three days. This was followed by prednisone at 1 mg/kg/day (50 mg/day) and cyclophosphamide infusions at 1 g/month for six courses. Then, the patient was given azathioprine at 150 mg/day combined with intensive physical therapy. This three-pronged treatment enabled the patient to stand upright again after five months, walk with limited autonomy for everyday activities, and achieve optimal recovery after seven months with slight, persistent superficial sensory disturbances.

**Figure 1:** Enlargement and expansion of the spinal cord at the cervicothoracic level up to T5, with a centro medullary topography measuring 12x8 axial diameters, extending over 201 mm in height (MRI T2 sequence, sagittal section)

**Table I:** Guillevin criteria

Alteration of general condition
Fever
Multineuritis
Inflammatory syndrome
Primary cardiomyopathy
Infiltrated purpura and/or subcutaneous nodules
Polyarthritis
Myalgia
Distal arteriopathy
Non-infectious orchitis
Vascular and/or glomerular nephropathy
Painful abdominal crises
High blood pressure
Central neurological accident
Recent severe asthma

Diagnosis = 5/15 criteria (sensitivity of 70. 6%, specificity of 92. 3%)

## Case 2

Ms. OMC, a 45-year-old Gabonese civil servant, presented in December 2023 with progressively worsening myalgia in her limbs, complicated by three months of quadriplegia. Prior to the onset of this condition, she had no history of trauma, fever, flu, medication use, traditional herbal remedies, or alcohol consumption. For the past four years, she had experienced episodes of spontaneously regressive, debilitating asthenia with postprandial, spasmodic abdominal pain that alternately responded to nonsteroidal anti-inflammatory drugs and proton pump inhibitors. Her medical history included episodes of spontaneous bruising of the lower limbs, migraines that had been present for nearly 20 years, and an episode of amaurosis in November 2020 that resolved spontaneously. She had never been hospitalized or undergone surgery. In terms of obstetrics, she reported three spontaneous miscarriages in the first trimester. She had no children and had experienced non-pregnancy-related amenorrhea for two months. Despite a weight loss of 6 kg, her general condition was stable with a body mass index of 23 kg/m^2^. She was afebrile, normotensive, and eupneic. A clinical examination revealed quadriplegia and absent osteotendinous, cutaneotendinous, cutaneotendineous, cutaneotendineous, and cutaneotendineous reflexes. Spontaneous hematomas were present on the front of the left knee and the upper third of the leg, as well as multiple bruises on the lower limbs, predominantly on the inner sides of the legs and thighs. Laboratory tests showed a white blood cell count of 12,750/mm^3^ with a predominance of neutrophils, hemoglobin level of 13.5 g/dL, normal platelet count, sedimentation rate of 35 mm/hr, and CRP level of 98 mg/L. The creatine phosphokinase, blood ionogram, fasting blood glucose, cholesterol and its subfractions, calcium, phosphorus, transaminases, prothrombin time, and activated partial thromboplastin time were normal. There was proteinuria with nitrites, but no hematuria or leukocytes. Serology tests for hepatotropic viruses (HBsAg and anti-HCV antibodies) and HTLV-1 were negative. *Borrelia burgdorferi* serology was positive for IgG. A lumbar puncture revealed clear, normotensive, paucicellular fluid with rare macrophages and no polynuclear cells, lymphocytes, or atypical cells. The direct and indirect Coombs tests, antinuclear antibodies, anti-native DNA, soluble nuclear antibodies, anticardiolipin antibodies (IgM and IgG), anti-beta 2 glycoprotein 1 antibodies, and ANCAs were negative. Circulating lupus anticoagulant was positive. The spinal MRI showed transverse myelitis extending from C2 to C6 longitudinally (Fig. 2). Abdominal and pelvic CT scans, as well as renal and digestive artery angiography, were normal. An electromyogram could not be performed due to lack of availability. Based on the presence of seven out of 15 Guillevin criteria (alteration of general condition, polyneuritis, subcutaneous nodules, myalgia, painful abdominal attacks, glomerular nephropathy, and inflammatory syndrome), the diagnosis of polyarteritis nodosa was made. The patient was treated with a methylprednisolone (Solumedrol^®^) bolus of 960 mg/day for three days, followed by corticosteroid therapy of 1 mg/kg/ day of prednisone (Cortancyl^®^) (i.e., 80 mg/day), cyclophosphamide (Endoxan^®^) infusions at a rate of one gram/month for six months, followed by oral medication and antiplatelet therapy at a dose of 100 mg/day and 20 mg/day of rosuvastatin, respectively, as well as intensive physical therapy. Due to her slow recovery, after three months of treatment, the patient was transferred to Europe for plasmapheresis and continued functional rehabilitation. After six months, she regained autonomy in all four limbs.

**Figure 2:** Extensive centromedullary hypersignal from C2 to C6 (T2 sequence, sagittal section)

## Discussion

PAN remains the prototype of systemic necrotizing vasculitis. Two decades ago, PAN was reported in 36 to 50% of hepatitis B cases, a disease endemic to sub-Saharan Africa [2]. In 2020, Africa accounted for 26% of global hepatitis B cases [11]. Given this context, one might wonder whether PAN is underdiagnosed in sub-Saharan Africa. Often, these are single reported cases. In a study of 27 cases of vasculitis in Senegal, Ndongo et al. found two cases of PAN associated with hepatitis B virus (HBV) [9]. During PAN, HBV induces a “type III” reaction, in which immune complexes are trapped in the vascular walls, causing vascular lesions [16,17]. HBs antigen and anti-HBs antibody immune complexes have been identified in these lesions. Due to HBV vaccination and improved hygiene conditions, the frequency of PAN due to HBV has decreased from 30% to 7%. Our observations show HBs antigen negativity without calling the PAN diagnosis into question.

We retained the PAN diagnosis based on the presence of six and seven Guillevin criteria. We did not use the ACR criteria for diagnosis because arteriography to search for aneurysms and a biopsy of a small-to medium-sized artery to search for polynuclear cells in the arterial wall are difficult to perform in our patients. Additionally, the ACR criteria have lower specificity (86% vs. 92.3%) (Table II).

**Table II:** Criteria of the American College of Rheumatology (ACR) 1990

Items	Value
Weight loss > 4 kg	1
Livedo reticularis	1
Testicular pain or tenderness	1
Diffuse myalgia, muscle weakness, or tenderness in the lower limbs	1
Mono- or polyneuropathy	1
Diastolic blood pressure > 90 mmHg	1
Renal failure (urea > 0. 40 g/L or creatinine > 15 mg/L)	1
Serum markers of hepatitis B (HBs antigen or anti-HBs antibodies)	1
Arteriographic abnormalities (aneurysms and/or occlusions of visceral arteries)	1
Biopsy of a small or medium-sized artery showing the presence of polynuclear cells in the arterial wall	1

Diagnostic = 3/10 critères (sensibilité de 82,2%, spécificité de 86,6%

Our patients have a history of migraine, miscarriages, and positive lupus anticoagulants, which are hallmarks of antiphospholipid syndrome (APS). They also present with transverse myelitis and major neurological deficits, such as quadriplegia and paraplegia, and their symptoms began during the dry season, which is the coolest time of year in Gabon.

APS is defined by a combination of obstetric and thrombotic symptoms accompanied by positive antiphospholipid antibody tests, including circulating lupus anticoagulant, anticardiolipin (IgM and IgG) antibodies, and anti-β2 glycoprotein-1 (IgM and IgG) antibodies [16]. Some authors have hypothesized that these antibodies promote the development of vasculitis by triggering vascular inflammation, which reveals cryptic antigens that stimulate anti-endothelial cell antibodies [5,10]. In some patients, these antibodies persist in the serum even when there is no evidence of disease activity [15].

The *vasa nervorum,* which supply blood to the peripheral nervous system, are one of the sites most frequently affected in PAN. The *vasa nervorum* penetrates the nerve and divides into complex, anastomotic microvascular networks that largely pass through the epineurium (the connective tissue that covers peripheral nerves) and fill the interfascicular space [1]. This could explain why up to 85% of PAN patients develop sudden-onset peripheral neuropathy with complete deficits (such as mononeuritis and polyneuritis) or partial deficits (such as ascending polyradiculoneuritis) [12]. Abnormalities of the central nervous system are less common than those of the peripheral nervous system and tend to occur later in the disease’s progression [7].

The rapid onset of paraparesis/paraplegia or tet-raparesis/tetraplegia, accompanied by a specific higher-level bilateral sensory deficit and sphincter disorders, defines acute total transverse myelopathies. In acute partial transverse myelopathies, spinal cord involvement is asymmetrical and sometimes unilateral; sensory deficit may be the primary symptom. Emergency spinal cord MRI is essential to rule out acute compressive myelopathy [6].

The occurrence of PAN during the coldest period of the year could prompt discussion about its role as a trigger for this disease’s expression. However, no seasonal pattern has been observed for this vasculitis in previous studies [13].

There are many etiologies of transverse myelitis. They are divided into primary causes, which are responsible for two-thirds of cases. Genetic data determine genetic susceptibility, and environmental factors trigger the disease. Secondary causes are responsible for one-third of cases. They include infectious causes, especially viral causes such as hepatitis B and C, HIV infection, parvovirus B19, and Epstein-Barr virus in its active form. Other causes include hematological causes such as hairy cell leukemia, myelomonocytic leukemia, and myelodysplastic syndrome. Autoimmune causes include lupus, antiphospholipid syndrome, multiple sclerosis, and optic neuromyelitis. In this case, a positive Coombs test and a sufficient number of Guillevin criteria pointed to an immunological cause, leading to a PAN diagnosis.

We observed slow neurological recovery in both patients, which took several months due to the axonal nature of the lesions. Recovery is often good, though sometimes incomplete with residual pain that may persist, as in one of our patients [14]. Innovative therapies, or biotherapies, have been used in cases of recurrent or refractory PAN. These include tocilizumab, anti-TNF alpha, and rituximab. They have resulted in complete remission in 50%, 40%, and 33% of cases, respectively. These therapies have comparable safety profiles [4]. In Gabon, the national health insurance system, also known as the Caisse Nationale d’Assurance Maladie et de Garantie Sociale (CNAMGS), covers 80% of the cost of medications for common conditions and 90% for long-term conditions [8]. Polyarteritis nodosa ranks 21^st^ out of 30 long-term conditions, alongside systemic lupus and systemic scleroderma. Currently, rituximab is the only biotherapy covered, and patients pay €145.30 for it.

## Conclusion

PAN is an autoimmune necrotizing vasculitis that is little known to African practitioners and can have a dire prognosis if left undiagnosed. It is important to raise awareness of this group of conditions in university medical programs in sub-Saharan Africa, and more generally of autoimmune diseases, which are mistakenly labeled by practitioners in sub-Saharan Africa as “Western diseases.”

## Patients’ consent

We have obtained the consent of the patients for the publication of this article.

## Funding

This study received no funding.

## Authors’ contributions:

IBA BA Josaphat: designer and writer MFOUMOU Annick: proofreader and data collector

KANGANGA EKOMY Arthur and Baderhwa Aurélie: bibliographic research, proofreaders TCHIBENET RAPOTCHOMBO Armel and SAPHOU DAMON Michel Arnaud: iconography selection, proofreaders

LEDAGA LENTOMBO Léonie Esther and IGALA Marielle: proofreaders

BOGUIKOUMA Jean Bruno: designer, proofreader, approver of the final version

## Conflicts of interest

The authors declare no conflicts of interest.
